# Death: Legal and Actual

**Published:** 1871

**Authors:** I. N. Danforth

**Affiliations:** Chicago


Article V.—*Death: Legal and Actual.* By I. N. Danforth, M.D., Chicago.

On the 26th of August, 1870, one J. H. Skaggs was hanged at Bloomfield, Stoddard Co., Mo. This cheerful scene "was public, and witnessed by more than twenty-five hundred persons;" every one of whom, we may add, with the exception of those necessarily present, ought to be thoroughly and everlastingly ashamed of themselves.

"At twelve minutes after one o'clock, the platform dropped, and the convict fell a distance of more than six feet. The noose was adjusted to the usual place, but at the drop the rope slipped behind the mastoid process. In three minutes all struggling ceased. At the end of four minutes I found, (says Dr. Robert J. Jackson, of Bloomfield, whose letter, published in the Journal of the Gynæcological Society of Boston, for June, 1871, I am quoting), a distinct fremitus passing over the region of the radial artery, which entirely ceased at the expiration of six and one-half minutes; at the end of four minutes more all signs of life had disappeared and the body was blue; I then pronounced him dead, in which opinion Dr. J. F. McDonald coincided. The body was allowed to hang four minutes longer; in all, fourteen and one-half minutes since the fall. I then suggested the removal, which was at once done."

The body was taken a distance of fifty yards to a room in the court house, and Drs. Jackson and McDonald "proceeded to the experiment of resuscitation." Artificial respiration failing to produce the desired effect, the poles of a galvanic battery were placed over the pneumogastric nerves, and strong currents were "passed into the body at intervals of four seconds; this was soon followed by indications of respiration." At this point, the sheriff interfered, and "ten or fifteen minutes were lost." The experiment was then resumed; but another interruption followed. "At eighteen minutes past two o'clock—writes Dr. Jackson—we again went to work; and in a few minutes the action of the heart and radial pulse were perceptible."

At this juncture the sheriff—who was evidently no baby in the matter of strategy—executed a brilliant flank movement by carrying off the wires belonging to the battery, and this interrupted the business for the space of thirty-eight minutes. "After recovering the wires and working a short time, Skaggs swallowed a small quantity of brandy and water." Cayenne pepper and whisky were employed to excite the circulation in the extremities; bleeding was resorted to—and, as a result of all this, the pupil contracted, "the pulse assumed a strong and steady action, and the breathing became easier and more regular. He now used the ocular muscles, his eyes following persons around the room. This was more marked at nine o'clock; at which time, by the interference of a mountebank, the opposition of the populace became so violent that we were obliged to relinquish all further experiment. Skaggs lived until four o'clock the next morning." (loc. cit.)

It is pleasant to be assured that at least a portion of the twenty-five hundred spectators maintained a lively interest in this performance up to its final termination: "the room was crowded almost to suffocation during the whole experiment."

In spite of the "wire pulling" of the sheriff, and of the "interference" of the "mountebank," and of the "opposition of the populace," Scaggs would undoubtedly have recovered, had the "proper remedies" been administered. Mr. C. S. Halsey, of Chicago, to whom a report of the case was sent, says that "arnica or perhaps belladonna should have been given at first, and when the pupil contracted, opium seemed indicated." Those physicians, therefore, who propose in future to rob the scaffold of its terrors will do well to take notice that arnica, belladonna and opium are "homœopathic" to hanging, and govern themselves accordingly. I may be pardoned for remarking, by the way, that the "provings" which demonstrated this fact, must form an interesting chapter in the history of homœopathic therapeutics.

Passing by the excessive barbarity of this whole performance on the part of the physicians concerned therein; passing by the weak and shuffling sheriff who permitted this odious business, which it was clearly his duty to arrest, *vi et armis*, if need be; passing by the gaping crowd of twenty-five hundred which stood idly by, neither of which particularly concern the readers of a medical journal, let us glance at a few points which *do* interest us as physicians, from a humanitarian, medical or medico-legal point of view.

From a humanitarian stand-point, this affair must be condemned as a most ingenious refinement of cruelty. If it shall ever occur to me to be half-hung by some blundering executioner, in the presence of twenty-five hundred people, I pray that Drs. Jackson and McDonald may not be there. I can conceive of no more grievous or terrible burden ; I can imagine no more bitter or horrible torture, than to be again invested with thought and memory after having been pronounced guilty by a jury, sentenced by a judge, and ignominiously hung by the neck, in answer to the law's demand. If the attempt be made to defend this resuscitation of Skaggs upon the ground of mercy, the obvious reply must be that it was mercy so disguised by cruelty, that the merciful element is crowded out of sight and hearing. Whatever humanity demands of the physician, it does *not* require that he shall step between the law and the criminal upon whom that law inflicts its penalty. The same principle carried out, would justify the physician in throwing open the prison doors, and, in fact, in practically bidding defiance to all attempts to enforce criminal law. Nor does science either require or sanction any such experiments upon human beings. If any such experiments are needed at all, they can just as easily and just as satisfactorily be performed upon the lower animals. In this case science may, with peculiar appropriateness, protest against being made the apologist for barbarism.

From a medical, or, more correctly, from a physiological standpoint, this case is one of peculiar as well as painful interest. Drs. Jackson and McDonald, being present as medical experts, pronounced Skaggs dead, and then immediately proceeded to demonstrate that he was *not* dead. As a matter of fact, profound asphyxia was mistaken for actual death, and it is highly probable that many criminals have been removed from the gallows under the sanction of medical authority, in like condition. Nor could this be esteemed a proper matter for criticism, even if one were so inclined, as I certainly am not. Two points, however, are of practical importance in this connection. The first is, that in many cases of death by hanging, the neck is not dislocated, and therefore death must occur by the comparatively slow and terribly painful process of strangulation. Criminals in America, who suffer the extreme penalty of the law, are the victims of much professional botch-work. If the case of Skaggs is any criterion, a criminal ought to be thankful who gets himself fairly and honestly executed even by strangulation.

The skill of Jack Ketch, who "worked off" the inmates of Newgate with such dexterity, perished with him, and those who propose to indulge in the luxury of murdering their fellow men, ought to take this fact into consideration beforehand.

In Skaggs' case, writes Dr. Jackson, "the noose was adjusted to the usual place"—wherever that may be—"but at the drop the rope slipped behind the mastoid process." Yet this case is neither the first nor the worst of its kind. In England the attempt was once made to save a criminal by making an incision into the trachea, below the rope, and introducing a tube to permit the continuance of respiration. "After being taken down, blood was drawn from the jugular vein, the malefactor opened his eyes and sighed, but did not recover." (Dean's Medical Jurisprudence, p. 440.)

Mr. Dean relates two other cases, which I quote as follows: one of a "woman in the reign of Henry VI, who remained suspended one whole night, and was still alive, owing, as it was said, to her larynx having been ossified;" and another "of a woman convicted in Edinburgh in 1728, who after having the sentence of the law executed upon her, or attempted so to be, recovered, and afterward lived twenty-five years." (loc. cit.)

The next point of practical interest to physicians who are called upon to attend executions for the purpose of deciding as to the occurrence of death, may here be brought forward; namely, the absence of signs of life does not necessarily prove that death has taken place. The heart's action may have ceased, the pulse may be imperceptible, and yet the victim may not have passed beyond the possibility of resuscitation. In the case of Skaggs "all signs of life had disappeared, and the body was blue;" and yet, under more favorable circumstances, the attempt at resuscitation would have been successful. It seems to me not only a very possible but a very probable thing that, in a vigorous healthy person, who suffers a sudden and violent death, cell-life, or, in older phraseology, "molecular life," continues for some time after all appreciable signs of life have completely ceased. As Carpenter so aptly states it—"somatic death" and "molecular death" do not occur together. (Human Physiology, p. 865.) Each cell possesses an independent life of its own; each cell is a sort of vital magazine, and is therefore capable of living, even though the heart has ceased to act, so long as it can procure the means of growth, and this depends upon fresh supplies of blood sent to the various cells by the heart. Those phenomena which are suddenly interrupted by execution, such as locomotion, circulation and respiration, are not of themselves *life*, but rather the results of life. The cessation of either or all of them, therefore, does not necessarily or immediately imply death. To state the actual fact in the plainest way, the mere act of hanging, however skillfully performed, does not *immediately* lessen the aggregate of vital force in the individual cells of any organ in the body; and as criminals are commonly executed, actual death only takes place after vitality has been literally choked out of the body. Hence, while it is indeed a terrible fact, it is by no means an inexplicable fact, that it is sometimes possible to resuscitate criminals, after all the ordinary phenomena of life are wholly extinct. A very important practical question presents itself here, namely, how can the occurrence of death be determined with absolute certainty? The ordinary negative signs, as we have seen in the case under consideration, and as experience under similar as well as dissimilar circumstances has often demonstrated before, are not always reliable. Moreover, the dictates of humanity as well as decency—especially at public executions—demand that the malefactor be removed from the scene of his disgrace as soon as possible. On the one hand, the humane physician would shrink from demanding that an execution be prolonged after the extinction of life had become reasonably certain, as indicated by the apparently total absence of signs of life. On the other hand, he has no business there, unless he proposes to perform his duty according to the full and exact intent of the law; and *this* means *actual* not *probable* death. From the very nature of the case, it follows that there *can* be no positive symptoms or signs, which shall clearly and certainly indicate when profound asphyxia (apparent death) has been replaced by actual death. The mere absence of signs cannot properly be called signs;—and this is a question to be decided by negatives alone. No test, which infallibly proves the absence of life, has, to my knowledge, been discovered. The medical expert is therefore reduced to the necessity—disagreeable and painful though it be—of insisting that the criminal remain suspended until there shall be no possibility of resuscitation. In doubtful cases, it seems to me that the presence or absence of muscular contractility, as determined by the electric test properly and skillfully applied, would aid in determining the question of apparent or real death—especially if the subject were previously vigorous and healthy. One point more deserves mention; the physician who is called to witness an execution, with the understanding that he shall be allowed to experiment upon the criminal subsequently, can scarcely be considered competent to perform his duties impartially. He attempts at once to execute and to thwart the law. When the sheriff promises the physician the opportunity for experimentation he offers a direct premium for the poorest possible services, and, more than that, he adds to an odious duty, a needless and inexcusable cruelty.

Considered in its medico-legal relations, the case of Skaggs is one of great as well as novel interest. According to Dr. Horatio R. Storer, of Boston—an authority of undoubted excellence in medical jurisprudence—if an executed criminal be formally pronounced dead and surrendered as such by the sheriff, if he be afterwards reanimated, he is entitled to his life, having technically fulfilled the requirements of the law. This may possibly stand the test of law—or rather of legal quibbling, unjustly dignified by the name of law—but it is very frivolous common sense. It amounts to saying that a man may be *legally* dead and *actually* alive. Under other, but far different circumstances, this sometimes is the case. If a man be sentenced to imprisonment for life, the law no longer recognizes him. He no longer possesses any legal rights, and therefore no longer needs the protection of law; hence his life *legally* ceases and determines with his incarceration. But the case of a resuscitated criminal is a far different one. If he regains his life, he also regains his liberty, and with it some of the powers which pertain to life and liberty. The law may indeed refuse him the highest privileges of citizenship, but the commission of fresh crimes will *compel* the law to recognize him. The commission of crime means the transgression of law; the transgression of law means punishment *by* law and according *to* law, and this secures to the criminal one of the highest privileges which the law can confer—namely, trial by jury; and I apprehend it would make little difference whether the accused had been previously asphyxiated or not, or if so, whether the asphyxia was produced by the hangman's noose, or by carbonic acid gas, or by tumbling into Chicago river. In point of inexorable fact, therefore, whatever the theory may be, no man can be legally dead, so long as he possesses the power to harm his fellow men. The criminal imprisoned for life, is legally dead *provided his sentence be literally executed;* but let him regain his liberty, either by escape or through the intervention of the pardoning power, and he instantly becomes legally alive—that is, amenable to law. When he regains the privileges of liberty he also reassumes the responsibilities of liberty.

If the position of Dr. Carroll of the *New York Medical Gazette*—that, when a criminal supposed to be dead shall "prove not to be dead," it will be the duty of the sheriff to "hang him over again,"—be not a correct position, it certainly deserves to be. If the law, which clearly demands *death*, can legally accept asphyxia in place thereof, then language becomes meaningless and powerless. If law can accept an *intention*—honest though it be—for an *actuality;* if it can accept the *intention* to fulfill its requirements for the *actual* fulfillment thereof, in one case, it can in all cases. Let this doctrine be once established, and we shall have a standing premium for all manner of shuffling trickery, to evade the literal demands of law. Let the doctrine be once established that the laws against crime may be executed according to the whimsical interpretations of men, rather than their strict and literal meaning, and society will be turned upside down. The law must say precisely what it means, and mean precisely what it says, and it must be executed by the actual and literal and perfect, not *intended*, performance of its requirements, or, instead of a government based upon law, we shall have anarchy and confusion. Again, if the position of Dr. Storer can be successfully defended, how can that sheriff justify himself who kills his victim outright hereafter? or how can that physician justify himself who witnesses and sanctions the absolute destruction of life on the gallows? In other words, if a good sound choking satisfies the law's demand, when accompanied by the usual formalities incident to hanging, do not the medical expert and the sheriff needlessly and wantonly destroy life when they do more than administer the choking? and if life be needlessly and wantonly destroyed, is it not likewise criminally destroyed? There is no escape from this conclusion, unless logic and law have parted company forever. Criminals cannot be legally hung and legally half-hung, in answer to the demands of precisely the same law, for the commission of precisely the same crime, nor can any useless formalities, or idle mummeries, performed by sheriff or physicians, bring falsehood out of fact, or fact out of falsehood.

But, admitting for a moment the logic upon which Dr. Storer's interpretation of the law is founded, a question of considerable practical interest—at least to the resuscitated criminal—presents itself; namely, the question of his own identity. If, by reason of having been asphyxiated under sanction of law, he is thenceforth to be regarded as one dead, he must of course lose his own personality as a member of society. If he loses his own identity, whose identity does he gain? Is he *himself* or somebody else? He must certainly buy at least food and raiment, and he must certainly sell the products either of his head or his hands, and these are transactions amazingly like those of persons not only alive but legally alive. By improving slightly upon the fiction of the law in both directions, however, we can very comfortably escape from our dilemma without in the least damaging its value or truthfulness—or falsity either, for the matter of that. If we substitute death for asphyxia, (following the example of the physicians who watched the demise (!) of Skaggs,) and resurrection for resuscitation, we shall thus find plain sailing, for a resurrected person can resume his former identity without permission of law—at least so far as experience has thrown any light upon the subject. Exactly how resurrections under such circumstances should be conducted, it is difficult to say, but probably "galvanism" and "bleeding," especially when aided by "cayenne pepper and whisky," will prove as effectual as anything. Precisely how resurrected criminals may be expected to conduct themselves we are still slightly in the dark, but it is more than likely that the most of them would naturally commence life anew by "swallowing a small quantity of brandy and water."

The case which I have considered stands not alone. The city of Philadelphia, with all its culture and civilization, has permitted a similar performance to be enacted, if my memory serves me correctly, in the amphitheater of the Jefferson Medical College. It would seem, therefore, that the luxury of experimenting upon the bodies of executed or half-executed criminals, is gradually coming to be regarded as a kind of perquisite, belonging to the physicians called upon to assist in executions. Such experiments are wrong in principle, unjustifiable and barbarous in practice, and without adequate benefit to science or humanity. On these grounds, then, they ought to be stopped at once and forever. Whatever may or may not be done to the mutilated body of the malefactor in a Christian land, one thing is certain: the medical profession ought to repudiate and sternly condemn a procedure so odious and disgusting, as the one which has given rise to this present article, and one which, if repeated, is so certain to bring deserved censure and odium, not alone upon those who are immediately concerned therein, but, to a greater or less extent, upon the profession as a whole.

